# Notes and Notices

**Published:** 1894-08

**Authors:** 


### NOTES AND NOTICES.

To Medical Publishers.—In consequence of the closing of the hotel at White Sulphur Springs, W. Va., the meeting-place of The American Medical Publishers' Association has been changed to Hot Springs, Virginia, which is but a few miles east of the place first selected, on the Chesapeake and Ohio Railway. The date has also been changed to Monday and Tuesday, August 13th and 14th. All medical publishers are cordially invited to attend, as matters of vital interest are to be discussed. Several interesting papers have been announced, and the meeting promises to be one that no publisher can afford to miss. All who contemplate attending this meeting will please advise the Secretary at once, stating the number of persons in the party, in order that provision may be made for their accommodation.

Charles Wood Fassett, *Secretary.*

The St. Louis *Clinique* has passed into the hands of Dr. Emory Lanphear, Professor of Surgery in the College of Physicians and Surgeons. Dr. Lanphear will conduct the journal in the interests of that school and of the medical profession of the West.

Dr. J. Emmons, of Richmond, Indiana, is just recovering from fracture of the hip. This accident, together with several complications, confined him to his room for the long period of three months. We wish the Doctor increased health and prosperity upon his return to professional duties.

Physicians in search of a place to settle in practice should read the offer in our advertising pages.

